# Femtosecond‐Laser Nanocavitation Regenerates SERS‐Active Plasmonic Nanogaps for Longitudinal Molecular Sensing at Biointerfaces

**DOI:** 10.1002/advs.76330

**Published:** 2026-07-03

**Authors:** Aditya Garg, Ze Zong, Meitong Nie, Stacie E. Deaver, Elizabeth M. Van Order, Elieser Mejia, Peter Vikesland, Erin S. Gloag, Wei Zhou

**Affiliations:** ^1^ Department of Electrical and Computer Engineering Virginia Tech Blacksburg Virginia USA; ^2^ Department of Mechanical Engineering Massachusetts Institute of Technology Cambridge Massachusetts USA; ^3^ Department of Biomedical Sciences and Pathobiology VA‐MD College of Veterinary Medicine Virginia Tech Blacksburg Virginia USA; ^4^ Department of Civil and Environmental Engineering Virginia Tech Blacksburg Virginia USA

**Keywords:** femtosecond‐laser nanocavitation, multiresonant plasmonics, sensor regeneration, surface enhanced Raman spectroscopy (SERS)

## Abstract

Longitudinal, long‐term molecular monitoring is critical for personalized medicine, yet protein adsorption in biofluids rapidly fouls bio‐interfaced sensors and restricts analyte access to sensing regions. Despite advances in antifouling coatings and regeneration strategies, repeated restoration of surface‐enhanced Raman spectroscopy (SERS) sensitivity in static, protein‐rich media remains difficult without degrading nanogap integrity, limiting longitudinal sensing. This work introduces a regenerative molecular sensor based on multiresonant plasmonic nanoprotruding meshes (MPNMs) that co‐localizes SERS sensing and nanocavitation‐based actuation within nanogaps anchored on a biocompatible polymeric mesh. The nanogaps are engineered to support an electric‐dipole resonance for SERS enhancement and a magnetic‐dipole resonance for photothermal conversion, enabling femtosecond‐laser‐triggered nanocavitation within SERS‐active nanogaps. Upon femtosecond‐laser irradiation, collapse of vapor nanobubbles (≈200 ns lifetime) generates thermomechanical forces that detach and displace foulants with micron‐scale precision to regenerate the nanogaps while preserving nanomorphology and optical performance. In undiluted human serum, regeneration restores detection limit for the Pseudomonas aeruginosa virulence factor pyocyanin from 2.0 µm to a clinically relevant 3.9 nm after 24 h of fouling. Repeated regeneration cycles enable spatiotemporal profiling of dynamic molecular signatures from P. aeruginosa biofilms in wound models over 24 h, establishing a self‐regenerating platform for longitudinal molecular monitoring in protein‐rich biosystems.

## Graphical abstract

A regenerative molecular sensing platform that co‐localizes surface‐enhanced Raman spectroscopy (SERS) sensing and nanocavitation‐based actuation. Femtosecond‐laser triggered nanocavitation produces thermomechanical forces to locally regenerate SERS‐active nanogaps in protein‐rich biofluids while preserving optical performance. Repeated regeneration cycles enable longitudinal SERS monitoring of bacterial biofilms within wound models over 24 h, establishing a self‐regenerating platform for long‐term molecular monitoring in protein‐rich biosystems.

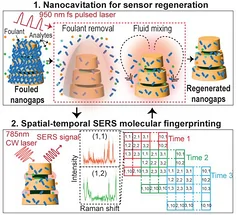

## 1 Introduction

Molecular sensing platforms capable of longitudinal analysis of dynamically changing biological fluids are emerging as next‐generation tools for personalized medicine and digital twin care paradigms [1, 2, 3, 4]. While these platforms offer the potential to detect early‐stage disease onset and inform timely therapeutic interventions, their realization remains constrained by severe physicochemical challenges. Traditional biosensors based on target‐receptor binding focus on a narrow set of biomarkers that limit diagnostic accuracy. In contrast, receptor‐free multi‐omics molecular sensing has demonstrated superior performance for complex disease diagnostics, such as cancer [5] and neurodegenerative disorders [6]. Despite this potential, achieving longitudinal, receptor‐free molecular sensing in biological systems remains a formidable challenge.

A representative and urgent clinical need for such technology is the management of chronic wounds, a condition affecting over 7 million individuals in the United States and incurring annual healthcare costs exceeding $25 billion [7]. A key contributor to chronicity is biofilm development, observed in over 80% of cases [8], in which bacterial communities embedded within an extracellular polymeric substance (EPS) matrix [9, 10] exhibit extreme antimicrobial tolerance (10–1000×) [11, 12]. Crucially, biofilms develop in a dynamic and spatially heterogeneous manner, driven by interdependent mechanisms like quorum sensing and metabolite secretion [13]. Thus, effective intervention necessitates longitudinal, in situ insights into these complex spatial‐temporal molecular dynamics that govern biofilm persistence and treatment resistance.

Surface‐enhanced Raman spectroscopy (SERS) is a powerful platform for such non‐invasive receptor‐free molecular sensing, combining molecular specificity with plasmonic signal amplification (10^4^ to 10^9^) in nanoscale hotspots [14, 15]. However, despite this potential, longitudinal operation in biological fluids is limited by biofouling, which rapidly blocks analyte access to SERS hotspots [16]. Passive strategies like zwitterionic coatings [17], sacrificial layers [18], and antifouling topographies [19] can only delay fouling; their performance degrades upon prolonged exposure due to environmental sensitivity (e.g., pH, ionic strength) [20, 21] and oxidative desorption of unstable anchoring chemistries [22]. These limitations underscore the critical need for on‐demand strategies that can actively regenerate the sensor surface.

Existing active regeneration methods fail to meet the requirements for longitudinal implementation and bio‐integration (Table S1). Conventional ex situ methods (e.g., plasma cleaning [23], ultrasonic agitation [24], and thermal annealing [25]) are highly invasive and incompatible with living interfaces. While in situ electrochemical regeneration shows promise [26], it relies on wired electrodes and oxidative potentials, requiring complex interconnects impractical for flexible devices and risking restructuring of nanoscale hotspots. Similarly, photocatalytic degradation [27] generates reactive oxygen species harmful to host tissue, while electrokinetic desorption [25, 28] is ineffective against neutrally charged biomolecules and in high‐ionic‐strength biofluids due to Debye screening. Critically, a major conceptual gap in prior in situ strategies is the lack of an active mass transport mechanism. Without directed fluid flow, desorbed molecules remain trapped within the viscous boundary layer and rapidly re‐adsorb onto the sensor surface. Therefore, a significant gap remains for a bio‐integrated regeneration technology that is contact‐free, preserves nanostructure integrity, and generates sufficient local forces to desorb foulants and actively transport them away from the interface.

Nanocavitation, the cyclic formation and collapse of nanoscale vapor bubbles, offers a compelling solution [29]. Attributed to physical phenomena such as fluid jetting [30] and ultrasonic shockwaves [31], cavitation can effectively remove foulants from surfaces [32, 33]. Plasmonic nanostructures enable nanolocalized photothermal heating via non‐radiative plasmon relaxation [34], allowing ultrashort‐pulsed laser excitation to generate transient temperature spikes that exceed the nucleation threshold and trigger nanocavitation [35]. Because cavitation can produce shear stresses surpassing 100 kPa near the bubble nucleation site [36, 37], the resulting mechanical forces can dislodge adsorbed foulants without damaging the surroundings. Uniquely this process induces transient fluid dynamics, specifically acoustic streaming [38] and convective thermosmosis [39, 40, 41] that act as a localized “pump.” This active transport mechanism can mix and propel desorbed molecules away from the sensor surface, distinguishing nanocavitation from methods where detached molecules can easily re‐adsorb in a static macroscopic environment. Despite these capabilities, which are highly desirable for biosensor regeneration, plasmonic nanocavitation has not yet been harnessed for restoring fouled sensing interfaces due to challenges in preventing structural degradation and spatially co‐localizing sensing and actuation.

In this work, we overcome these barriers by introducing multiresonant plasmonic nanoprotruding meshes (MPNMs). MPNMs are biocompatible polymeric meshes containing nanolaminated plasmonic nanogap arrays that co‐localize nanocavitation and SERS within the same mechanically‐stabilized nanogaps. By engineering hybrid localized surface plasmon modes, specifically an electric dipole (ED) mode at 785 nm for SERS and a magnetic dipole (MD) mode at 950 nm for heating, we achieve both strong SERS enhancement and efficient photothermal transduction. We show that fs‐laser‐induced nanocavitation reliably regenerates these SERS‐active nanogaps with micron‐scale spatial precision. Following 24 h of exposure to human serum, this regeneration restores the detection of pyocyanin, a secreted *Pseudomonas aeruginosa* virulence factor [42], to a clinically relevant limit of detection (LoD) of 3.9 nm, representing a three order of magnitude improvement over non‐regenerated controls. Finally, we leverage this capability to achieve regenerative, spatiotemporal SERS monitoring of *P. aeruginosa* biofilms over 24 h in an in vitro wound model, capturing dynamically secreted components (e.g., EPS, virulence factors) otherwise inaccessible without regeneration.

## 2 Results and Discussion

### 2.1 Design and Fabrication of Multiresonant Plasmonic Nanoprotruding Meshes (MPNM)

To fabricate the MPNMs, we employed a high‐throughput nanofabrication process comprising three key steps: (1) transferring nanohole array patterns onto lithographically defined polymeric meshes via soft nanoimprint lithography, (2) depositing alternating layers of gold and SiO_2_, followed by resist stripping and selective SiO_2_ etching to define vertically‐stacked nanolaminated plasmonic nanogaps, and (3) generating protruding nanopillars through reactive ion etching, using the nanostructures themselves as an etch‐protective mask (Figure S1 and methods). Figure 1A shows an image of the MPNM interfaced with an in vitro wound model, alongside a bright‐field image revealing the micro‐porous mesh and a top‐down scanning electron microscopy (SEM) image showing the ordered array of plasmonic nanostructures. Tilted‐view SEM and focused ion beam‐SEM (FIB‐SEM) images reveal the protruding nanopillar arrays that contain the vertically‐stacked, nanolaminated plasmonic nanogaps mechanically stabilized through adhesion layers between each stack. Figure 1D schematically illustrates the key mechanical attributes of the MPNMs that enable long‐term, wound‐interfaced biofilm monitoring: (a) a porous architecture that permits oxygen exchange [43, 44], (b) high flexibility with protruding nanopillars carrying plasmonic nanogaps, allowing conformal interfacing with irregular wound surfaces and penetration into the biochemically rich wound environment, and (c) tissue‐like mechanical properties and biocompatible materials that support stable, long‐term bio‐integration (Text S1) [45].

**FIGURE 1:**
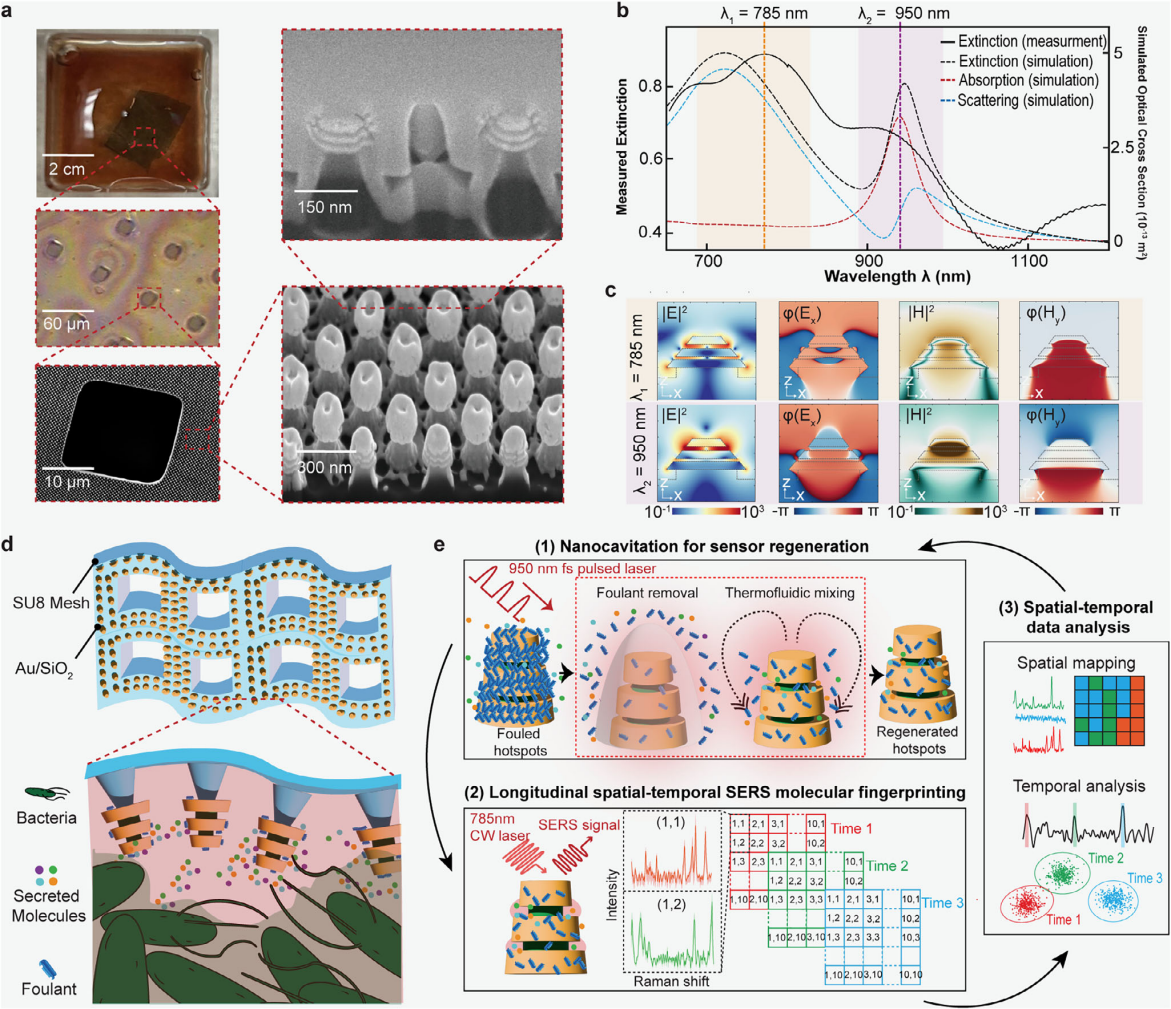
Multiresonant plasmonic nanoprotruding meshes (MPNM) for longitudinal, spatial‐temporal SERS molecular fingerprinting at biological interfaces. a) Images of the MPNM at multiple scales. Top row: A camera image shows the MPNM interfaced with an in vitro wound model, alongside a bright‐field image revealing the micro‐porous mesh and a top‐down scanning SEM showing the array of square nanogaps. Bottom row: Tilted‐view SEM and FIB‐SEM images reveal the protruding nanopillar arrays that house the vertically stacked, nanolaminated plasmonic nanogaps. b) Optical properties of the MPNM. FDTD‐calculated absorption, scattering, and extinction spectra along with the experimentally measured extinction spectra, confirming the engineered resonant modes at the SERS excitation wavelength (785 nm) and the fs laser nanocavitation actuation wavelength (950 nm). c) FDTD calculated maps of |E|^2^, f(E_x_), |H|^2^, and f(H_y_) at 785 and 950 nm. d) Schematic of the MPNM, highlighting key features for seamless bio‐integration: a porous structure enabling mass transport and a conformal nano‐bio interface designed to intimately contact the wound bed. e) Workflow for regenerative spatiotemporal SERS sensing. The proposed pipeline integrates three key stages: (1) sensor regeneration, where a pulsed laser induces nanocavitation to remove biofouling while thermoplasmonic flow facilitates molecular mixing; (2) Spatiotemporal SERS fingerprinting, where a continuous‐wave (CW) laser probes molecular signatures at different times and locations; and (3) Spatiotemporal data analysis, where machine learning is used to extract actionable insights.

Using finite‐difference time‐domain (FDTD) simulations, we designed the nanolaminated plasmonic nanogaps to support wavelength‐multiplexed operation: SERS at 785 nm and plasmonic nanocavitation at 950 nm (Figure 1B). The 785 nm resonance exhibits an ED character, as indicated by the in‐phase electric fields (ϕ(E_x_)) across the metal layers (Figure 1C), efficiently coupling to free‐space radiation with minimal absorptive loss (Figure 1B). This scattering‐dominated mode produces intense electromagnetic confinement within the nanogaps, yielding strong local field enhancement (∣*E*∣^2^ > 10^3^) (Figure 1C), that drives strong SERS amplification. Experimentally, this design achieved an exceptional SERS enhancement factor of 10^7^ at 785 nm (Figure S2). In contrast, the 950 nm resonance displays a pronounced MD character, arising from out‐of‐phase electric fields (ϕ(*E*_x_)) in the top metal layers that generate circulating displacement currents and intense magnetic fields (∣*H*∣^2^) within the nanogap (Figure 1C). The MD mode is weakly radiative due to destructive interference of the out‐of‐phase fields, resulting in suppressed scattering and enhanced absorption (Figure 1B). Consequently, most of the coupled optical energy is dissipated through Ohmic losses, leading to efficient photothermal conversion. Importantly, this mode also sustains comparable near‐field enhancement (∣*E*∣^2^ > 10^3^), ensuring strong nanogap confinement while maximizing thermal energy generation, ideal for nanocavitation actuation. The experimental extinction spectra of the MPNMs are in close agreement with simulated responses, confirming the successful realization of spatially overlapped ED and MD plasmonic modes at 785 and 950 nm, respectively, on a biocompatible polymeric scaffold. This engineered dual‐mode response establishes the foundation for bio‐integrated multimodal operation, integrating SERS sensing with plasmonic nanocavitation.

Figure 1E shows our experimental workflow. In complex wound environments, rapid fouling of plasmonic metal surfaces by biomacromolecules can impede analyte diffusion to SERS hotspots within hours. We hypothesize that under fs pulsed laser excitation, nanocavitation will remove fouling layers via thermomechanical processes, while thermofluidic processes will promote mixing between desorbed species and surrounding molecules, thereby refreshing the sensing environment. Collectively, these surface‐cleaning and fluid‐mixing phenomena will regenerate SERS‐active nanogaps prior to each SERS measurement. By applying multivariate analysis to the spatiotemporal SERS datasets, embedded with both spatial and temporal molecular signatures, meaningful insights into dynamic biofilm behavior can be extracted to guide personalized therapeutic interventions.

### 2.2 Plasmon‐Induced Nanocavitation for SERS‐Active Nanogap Regeneration

As nanocavitation relies on the cyclic formation and collapse of vapor nanobubbles, elucidating the underlying bubble dynamics is critical for controlling the regeneration process. Using a custom‐built pump‐probe setup, we experimentally characterized these dynamics on the MPNM under 950 nm fs laser irradiation. When immersed in water, the MPNM is irradiated by a fs pump laser (950 nm, 0.5 MHz, 0.09 nJ µm^−2^ fluence) to generate nanobubbles, while a colinear continuous‐wave (CW) probe laser (780 nm) monitors the back‐scattered signal to measure bubble lifetimes (Figure 2A). Further experimental details are provided in the Methods section and Table S2. Fast Fourier transform analysis of the photodetector signal in the frequency domain revealed peaks at harmonics of the pump laser's fundamental repetition rate, confirming light scattering by vapor bubbles on the plasmonic surface (Figure 2B). The corresponding reconstructed temporal signal showed a ≈200 ns signal drop caused by scattering losses, reflecting the transient formation and collapse of nanobubbles and establishing a bubble lifetime of ≈200 ns.

**FIGURE 2:**
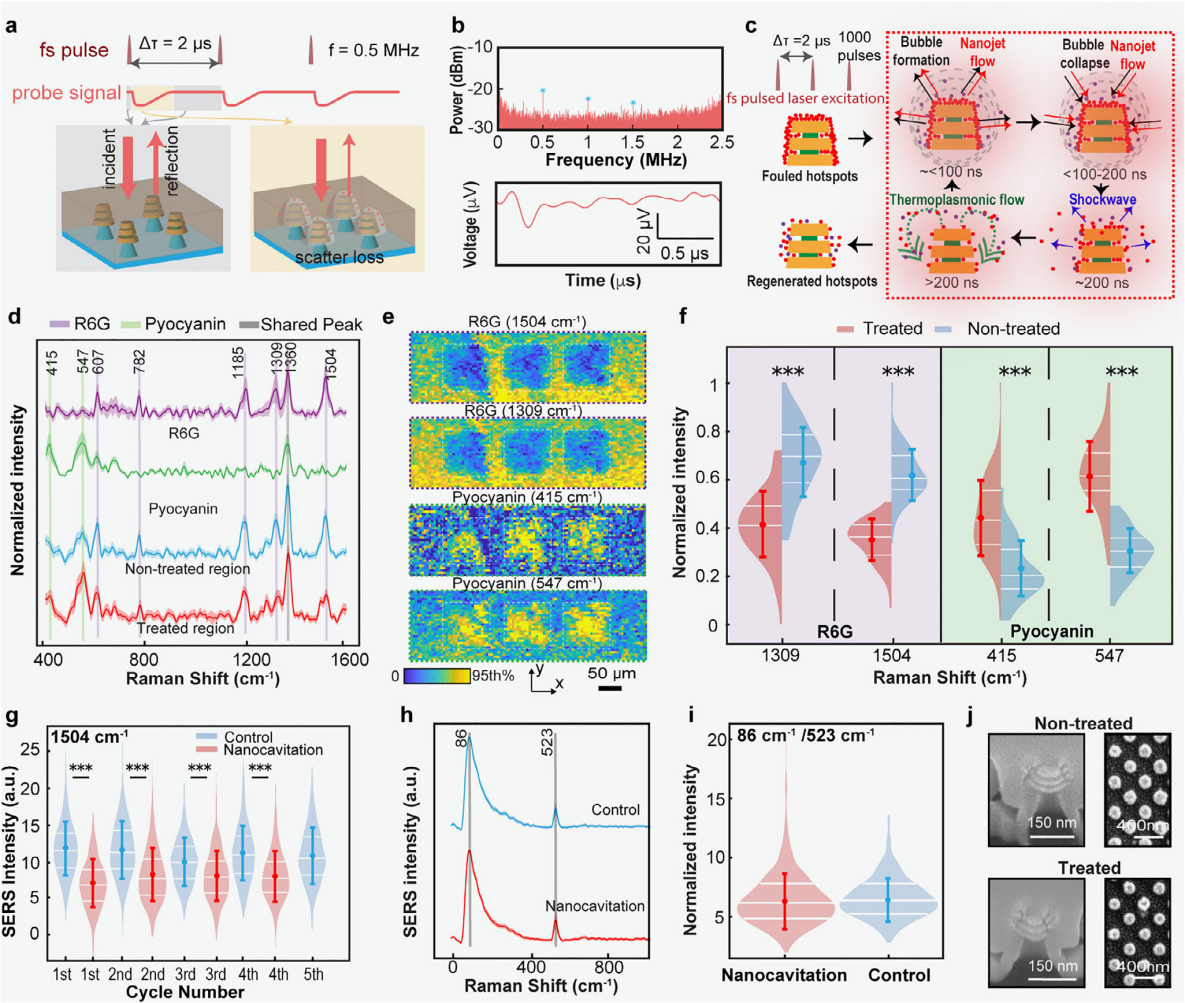
Plasmon‐induced nanocavitation for SERS‐active nanogap regeneration. a) Schematic of the experimental setup used to characterize plasmon‐induced vapor nanobubbles. The MPNM is irradiated with a fs pump laser (950 nm) to generate nanobubbles, while a colinear CW probe laser (780 nm) monitors the back‐scattered signal to measure their dynamics. b) Validation of nanobubble generation. The fast Fourier transform of the photodetector signal reveals distinct peaks at harmonics of the pump laser's repetition rate. The corresponding reconstructed temporal signal shows a ≈200 ns signal drop, confirming the transient formation and collapse of vapor nanobubbles. c) Conceptual schematic of the hotspot regeneration process. Under fs‐pulsed laser irradiation, cyclic nanobubble formation and collapse generate thermofluidic flow and shockwaves. This process mechanically dislodges an initially adsorbed analyte (R6G), enabling a second analyte (pyocyanin) from the solution to access the regenerated hotspots. d) SERS spectra of nanocavitation treated versus untreated controls. Shaded regions represent standard deviations. The data show diminished R6G peaks (purple) and enhanced pyocyanin peaks (green) after nanocavitation treatment. e) SERS intensity maps of the R6G peaks (1504 and 1309 cm^−^^1^) and the pyocyanin peaks (415 and 547 cm^−^^1^), with the white box indicating the nanocavitation treatment region. The maps show a localized decrease in R6G signal and a corresponding increase in pyocyanin signal within the treated regions, demonstrating spatially confined regeneration. f) Quantitative comparison of analyte signals showing a statistically significant decrease in R6G peak intensities (1504 and 1309 cm^−^^1^) and an increase in pyocyanin peak intensities (415 and 547 cm^−^^1^) in treated versus non‐treated regions (***p < 0.001 (two‐sample t‐test); error bars represent standard deviation; *n* = 75). g) Repeatability of the regeneration process. The intensity of the 1504 cm^−^^1^ R6G peak is shown across five consecutive cycles of fouling and nanocavitation‐based regeneration on the same spot, demonstrating high repeatability (***p < 0.001 (two‐sample t‐test); error bars represent standard deviation; *n* = 891–900). h) SERS spectra of a bare MPNM before and after five cycles of nanocavitation treatment, showing the plasmonic electronic Raman scattering (ERS) pseudopeak (86 cm^−^^1^) and the underlying silicon substrate peak (523 cm^−^^1^). Shaded regions represent standard deviations. i) Preservation of plasmonic performance. The normalized ERS/Si peak intensity ratio remains consistent before and after five regeneration cycles, indicating that the plasmonic enhancement of the substrate is not degraded by the treatment (error bars represent standard deviation; *n* = 742–778). j) SEM and FIB‐SEM images of the MPNM nanogaps before treatment and after five cycles of treatment, revealing no discernible morphological changes and confirming the structural robustness of the substrate.

We hypothesize that nanocavitation drives surface cleaning through a synergistic coupling of nanolocalized thermal desorption and mechanical disruption (Figure 2C). Fundamentally, localized plasmonic heating raises the surface temperature and accelerates molecular desorption through a thermally activated pathway described by Arrhenius kinetics, wherein the desorption rate increases exponentially as more adsorbates acquire sufficient energy to exceed the activation barrier [46, 47, 48]. Because fs pulses generate nanolocalized plasmonic heating with ultrafast heat dissipation within nanoseconds, heat exposure to the surrounding biological environment remains minimal [49, 50]. In parallel to this thermal destabilization, nanocavitation generates high‐speed liquid jets and shockwaves that impose shear stresses exceeding 100 kPa near the bubble nucleation site [36, 37]. These intense mechanical forces disrupt interfacial bonds and eject bound molecules. We propose that plasmonic heating and nanocavitation‐induced mechanical forces act synergistically within the 200 ns bubble lifetime to provide sufficient energy for foulant desorption from plasmonic interfaces. Under fs‐pulsed train excitation, these cavitation events recur ≈1000 times at a 2 µs repetition period, progressively weakening complex surface interactions and eroding foulant layers to restore the SERS‐active nanogaps. Moreover, transient fluid motion during nanocavitation [39], followed by sustained thermally induced flows such as convection and thermososmosis [40, 41], can enhance mixing between desorbed species and the surrounding medium. This dynamic fluid environment reduces the likelihood of re‐adsorption and further supports efficient clearance of interfacial contaminants.

To test this hypothesis, we performed a dual‐analyte experiment. A droplet of 1 mm rhodamine 6g (R6G) solution was evaporated onto the MPNM and rinsed four times with water to remove weakly bound molecules. R6G can strongly adsorb to gold via π–metal interactions [51] with an adsorption free energy of ≈110 kJ mol^−1^ [52], restricting other analytes from accessing the nanogaps. Afterward, a second analyte, 10 µm pyocyanin solution, was introduced. Then, nanocavitation was induced to regenerate the nanogaps in three predefined regions (100 µm × 100 µm) by raster scanning with a fs laser (1000 pulses at 2 µs repetition rate per spot, 0.09 nJ µm^−^^2^ fluence) and SERS images were collected. Figure 2D presents the SERS spectra from both fs‐laser‐treated and untreated regions. In treated regions, R6G peak intensities (607, 782, 1185, 1309, and 1504 cm^−^^1^) decreased markedly, indicating effective removal of R6G from nanogap hotspots. Concurrently, pyocyanin peaks (415, 547 cm^−^^1^)) increased in intensity, reflecting improved molecular access to regenerated hotspots (Figure 2D). Raman mapping of R6G (1309 and 1504 cm^−^^1^) and pyocyanin (415 and 547 cm^−^^1^) further confirmed localized regeneration with micron‐scale precision, showing decreased R6G and increased pyocyanin signals within laser‐treated areas (Figure 2E). This spatial confinement is critical for in vivo applications, where preservation of the surrounding environment is essential. Notably, the most pronounced regeneration appeared at the centers of the treated regions, consistent with nanocavitation events in adjacent nanogaps contributing to the cumulative cleaning effect. Quantitative analysis confirmed statistically significant increases in pyocyanin peak intensities (415 and 547 cm^−^^1^)) accompanied by significant reduction in R6G peaks (1309 and 1504 cm^−^^1^) (*p* < 0.001 for all compared peaks), validating the nanocavitation‐based regeneration approach (Figure 2F).

To decouple the contributions of thermal effects and nanocavitation to nanogap regeneration, we repeated the dual‐analyte experiments using fs laser fluences below the bubble‐generation threshold (0.03 nJ µm^−^^2^) and above the threshold (0.09, 0.105, and 0.12 nJ µm^−^^2^) (Figure S3). Bubble generation was confirmed through pump‐probe measurements. At fluences above the bubble threshold, the pyocyanin peak intensity increased by ≈2.1‐fold relative to the sub‐threshold condition, while the R6G peak intensity decreased by ≈1.9‐fold, indicating a substantial improvement in regeneration performance upon the onset of nanocavitation. Notably, no significant further increase in pyocyanin intensity or decrease in R6G intensity was observed as the laser fluence was increased from 0.09 to 0.12 nJ µm^−^^2^, despite the greater thermal energy deposited at these higher fluences. These results suggest that nanogap regeneration is strongly driven by mechanical forces generated during nanocavitation rather than by thermal effects alone (see Text S2 for a detailed discussion).

To evaluate the cycle‐to‐cycle repeatability, we performed five consecutive iterations of R6G detection and nanocavitation‐mediated regeneration on the same micro‐region. The intensity of 1504 cm^−^^1^ R6G peak consistently modulated between fouled and cleaned states, yielding a relative standard deviation of 5.77% and 7.72% across the mean intensities in the fouled and cleaned states, respectively. Furthermore, the standard deviation of the SERS maps remained tightly bounded across all regeneration cycles, varying from 3.1777 to 3.8799, corresponding to a ≈19.9% variation relative to the mean standard deviation (≈3.53) (Table S3). No monotonic trend was observed with increasing regeneration cycles, indicating that repeated fs‐laser nanocavitation does not induce progressive deterioration of spatial uniformity in the SERS response. To rigorously validate the functional integrity of the nanogaps, we analyzed the plasmonic electronic Raman scattering (ERS) signal. The ERS pseudopeak at 86 cm^−^^1^, normalized to the characteristic silicon substrate peak at 523 cm^−^^1^ (Figure 2H). The ERS signal, arising from surface‐plasmon‐enhanced inelastic scattering of sp‐band electrons [53], serves as an intrinsic proxy for plasmonic enhancement. The normalized ERS intensity remained consistent before and after regeneration, indicating preserved plasmonic performance throughout the cycling (Figure 2I). Finally, SEM and FIB‐SEM imaging confirmed that the nanogap hotspots remained structurally intact with no discernible morphological degradation, further confirming the structural robustness of the substrate (Figure 2J).

### 2.3 Regenerative SERS Detection of Pyocyanin after Incubation in Human Serum

To demonstrate the performance of our platform in blood, we determined the LoD of pyocyanin, a key virulence factor secreted by the opportunistic pathogen P. aeruginosa, in undiluted human serum after 24 h incubation with the MPNM. This experiment replicates realistic physiological conditions in which the device would be exposed to blood prior to biofilm formation and metabolite release (Figure 3A). To minimize fouling, the MPNM was functionalized with a monolayer of L‐cysteine (Figure S4) [54]. At physiological pH, this naturally occurring amino acid is zwitterionic, possessing both positive (amino) and negative (carboxyl) charges (Text S3). This facilitates the formation of a tightly bound hydration layer through strong electrostatic interactions and hydrogen bonding with surrounding water molecules, hindering protein adsorption [55, 56]. L‐cysteine‐functionalized gold surfaces have shown reduced bovine serum albumin adsorption by ≈90% after 24 h incubation compared to uncoated controls. Despite its antifouling properties, L‐cysteine does not completely prevent biofouling during prolonged exposure to protein rich biological media. Thiol‐based monolayers are known to undergo gradual degradation in biological media through Au‐S bond exchange and oxidative desorption [57, 58], resulting in defects and progressive protein adsorption over time. Previous studies have reported the formation of protein coronas several nanometers thick on L‐cysteine functionalized gold nanoparticles within hours in bovine serum albumin [59], which can restrict access to the sub‐10 nm nanogaps that generate strong SERS enhancement. This underscores an inherent limitation of passive antifouling coatings and the need for active regeneration to restore nanogap accessibility and maintain ultrasensitive sensing performance.

**FIGURE 3:**
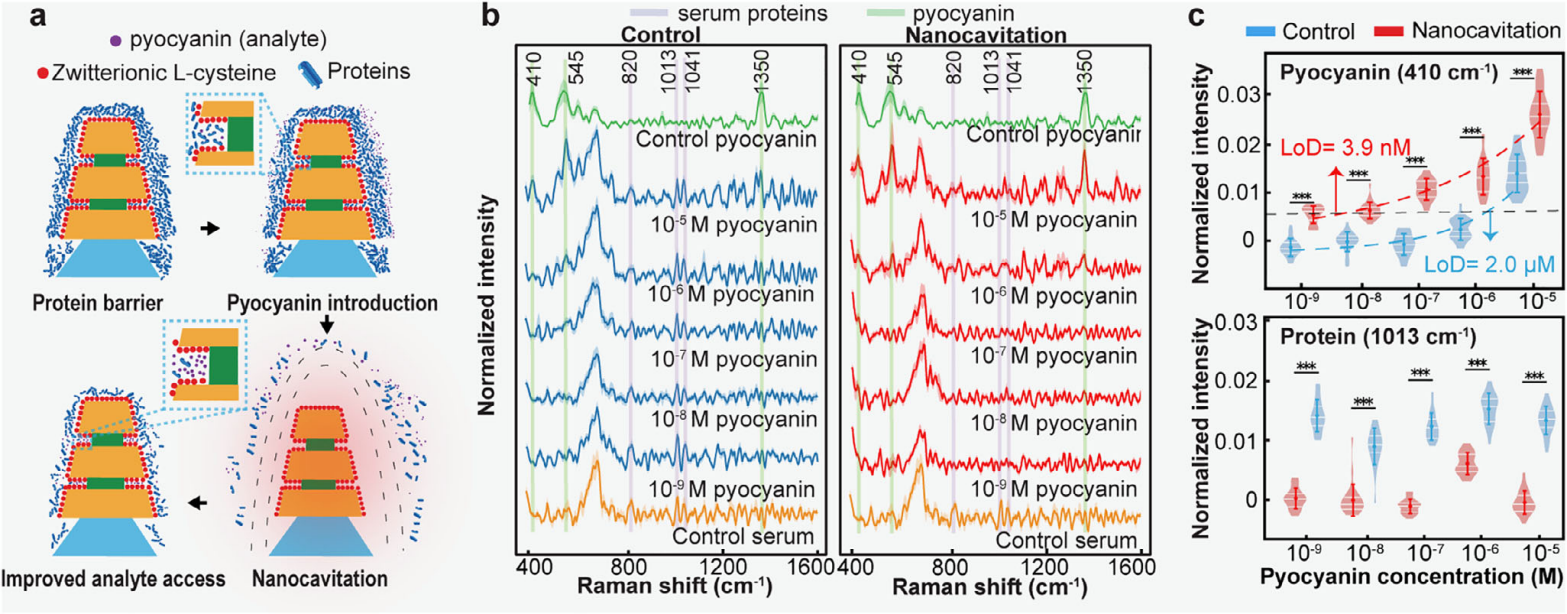
Regenerative SERS detection of pyocyanin after long‐term incubation in human serum. a) Schematic of SERS hotspot regeneration in a protein‐rich environment. Initially, a protein corona forms on the MPNM surface, blocking analyte access. Nanocavitation treatment removes this fouling layer, enabling subsequent detection of the target analyte (pyocyanin). b) SERS spectra comparing nanocavitation‐treated and untreated control samples after a 24‐hour incubation in human serum, followed by the introduction of pyocyanin at concentrations from 10^−^^9^ to 10^−^^5^ m. Shaded regions represent standard deviations. After treatment, characteristic pyocyanin peaks (green) become prominent, while serum protein peaks (purple) are significantly suppressed. c) Quantitative analysis of SERS signals. Violin plots show the 410 cm^−^^1^ pyocyanin peak and the 1013 cm^−^^1^ serum protein peak for the nanocavitation‐treated samples and untreated controls across a range of pyocyanin concentrations (error bars represent standard deviations; *** *p*  < 0.001 (two‐sample t‐test); *n* = 25; the red and blue dotted lines represent the four‐parameter sigmoidal fit for calibration). The data demonstrate increased pyocyanin signals and suppressed protein signals in the nanocavitation‐treated samples (pyocyanin LoD = 3.9 nm) compared to the untreated controls (pyocyanin LoD = 2.0 µm).

After 24 h serum exposure, pyocyanin was introduced at concentrations spanning 10^−5^–10^−9^ m. The substrate was raster‐scanned over a 4 mm^2^ area with a fs laser to induce nanocavitation, thereby regenerating SERS‐active nanogaps and enabling pyocyanin access to them. Figure 3B shows representative SERS spectra from nanocavitation‐treated and untreated controls. Distinct pyocyanin peaks are observed at 410, 545, and 1350 cm^−^^1^ in both cases. In the control spectra, strong protein‐associated peaks (e.g., 820, 1013, 1041 cm^−^^1^) dominate, but these are markedly suppressed following nanocavitation treatment (Table S4). Figure 3C and Figure S5 quantify the relative intensities of pyocyanin (410, 1350 cm^−^^1^) and protein (820, 1013 cm^−^^1^) peaks. The substantial reduction in protein signals confirms effective removal of adsorbed serum proteins, while the concurrent increase in pyocyanin peak intensity indicates enhanced molecular access to regenerated hotspots. A four‐parameter sigmoidal fit applied to the pyocyanin calibration curve (Figure 3C) yielded excellent *R* ^2^ values of 0.92 for both samples. Based on this calibration curve, the LoD for pyocyanin was determined to be 3.9 nm for the nanocavitation‐treated samples, compared with 2.0 µm for untreated controls. The pyocyanin LoD for untreated controls without L‐cysteine functionalization was 21.8 µm (Figure S6). LoD is defined as the concentration corresponding to the mean signal of the blank plus three times its standard deviation. This three order of magnitude improvement in sensitivity enables detection of pyocyanin at the lower end of clinically relevant concentrations (3–25 nm) reported in P. aeruginosa‐infected wounds, underscoring the potential for early‐stage infection diagnostics [60].

### 2.4 Regenerative Spatiotemporal SERS Monitoring of *P. aeruginosa* Biofilms in an In Vitro Wound Model

To better mimic the wound environment, we evaluated the long‐term spatiotemporal SERS fingerprinting of P. aeruginosa biofilms using an in vitro wound model (Figure 4A). The agar block biofilm assay (ABBA) [61] was modified for this model. Wound‐like media [62]. was solidified with 1% low melting point agarose. While molten, the media was inoculated with *P. aeruginosa* and transferred to an eight‐well chamber slide. Visualization by confocal laser scanning microscopy revealed spatially distinct aggregates form within the agarose matrix: larger biofilm clusters develop closer to the agar–air interface, where oxygen is abundant, while smaller aggregates arise deeper in the gel (Figure 4A), consistent with previous reports using the ABBA model [61]. Such non‐attached biofilm aggregates are dominant in most chronic wounds [63, 64], effectively replicating realistic growth dynamics. The MPNM was introduced topically at 0 h, and non‐invasive spatiotemporal SERS measurements were conducted every 6 for 24 h. By 12 h, bright‐field imaging revealed biofilm aggregates directly interfacing with the MPNM surface (Figure 4A).

**FIGURE 4:**
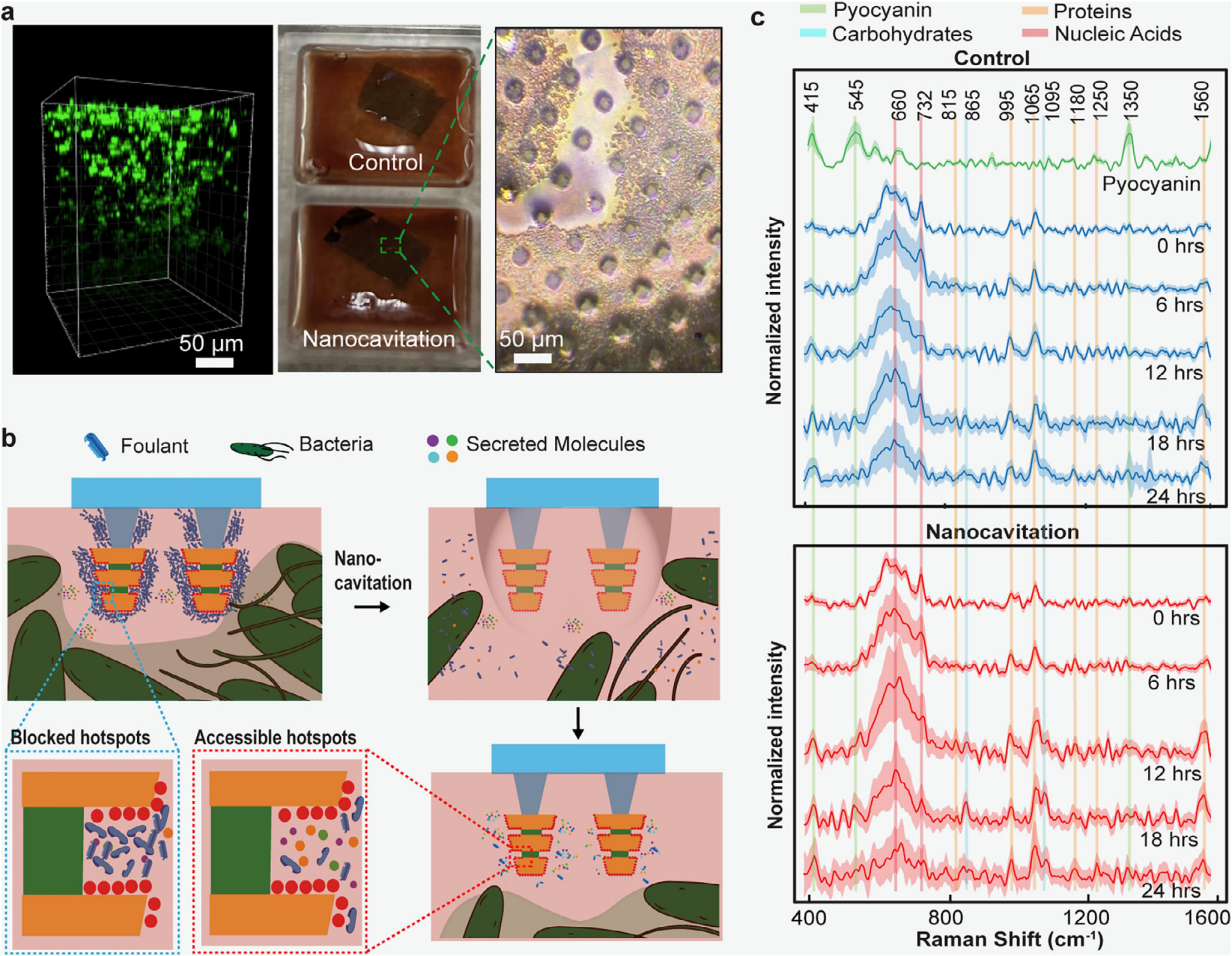
Regenerative spatiotemporal SERS monitoring of *P. aeruginosa* biofilms in in vitro wound models. a) Schematic of nanocavitation‐enabled regenerative SERS monitoring within the in vitro model. The MPNM, embedded in the in vitro model, becomes fouled by plasma protein and other extracellular components. Nanocavitation regenerates the SERS‐active nanogaps, facilitating the detection of dynamically secreted biomolecules like pyocyanin. b) *P. aeruginosa* biofilm growth in the ABBA model. A 3D fluorescence image shows biofilm aggregates suspended in the agar gel. A camera image displays the MPNM integrated with the ABBA model, and a bright‐field micrograph at 12 h reveals biofilm aggregates interfacing with the MPNM surface. c) Time‐resolved SERS spectra from 0 to 24 h of biofilm growth from three biological replicates. The spectra show the emergence of characteristic peaks from pyocyanin (green), proteins (orange), and nucleic acids (red) over time. Shaded regions represent standard deviations.

Our previous work demonstrated that for MPNMs interfaced with P. aeruginosa lawn biofilms grown on nutrient agar plates, strong pyocyanin peaks appeared within 12 h and increased with time [54], indicating that regeneration was unnecessary in this simplified medium (Figure S7). In contrast, the in vitro wound model contains high concentrations of serum proteins (e.g., albumin) and cellular components (e.g., red blood cells), which rapidly foul nanogap hotspots. To overcome this, fs laser nanocavitation was performed prior to each measurement for hotspot regeneration, with untreated controls included for comparison. Regeneration should allow dynamic access of freshly secreted molecules from the biofilm to the nanogap hotspots for accurate spatiotemporally‐resolved measurements (Figure 4B).

Figure 4C shows SERS spectra from 0 to 24 h, alongside a 10 µm pyocyanin reference spectrum. At 0 h, both nanocavitation‐treated and control samples displayed peaks from wound‐like media components (660, 682, 732, 995, 1065 cm^−^^1^) (Figure S8). By 12 h, nanocavitation‐treated samples exhibited pyocyanin peaks (415, 545, 1350 cm^−^^1^), along with features assigned to protein components such as tyrosine (815, 1180 cm^−^^1^), amide III (1250 cm^−^^1^), and amide I (1560 cm^−^^1^), and to carbohydrates (1095, 865 cm^−^^1^) (Table S4). These signatures reflect biofilm‐secreted extracellular metabolites (e.g., pyocyanin) [65, 66], and EPS matrix, which is composed primarily of polysaccharides, proteins, and extracellular DNA [67]. For instance, the polysaccharide backbone peaks at 1095 and 865 cm^−^^1^, corresponding to glucosidic ring vibrations [68], likely originate from Psl and Pel in our non‐mucoid P. aeruginosa strain [69]. These bands have been reported as prominent Raman features of P. aeruginosa EPS [70].

Because the spectra at different growth stages contain overlapping bands from diverse biomolecules, visual feature selection is challenging. We therefore applied principal component analysis (PCA) to extract the spectral features driving temporal variance. In control samples, PCA scatter plots showed substantial overlap between the 6 and 12 h time points, with clearer separation only emerging at 18 and 24 h (Figure 5A). In contrast, nanocavitation‐treated samples displayed distinct clustering at 6, 12, 18, and 24 h, indicating improved detection of dynamically secreted molecules driving separation, particularly during early biofilm development when concentrations of extracellular secretions are low (Figure 5B). The principal component loading plots reveal the spectral variables with strongest contributions to the variance. The first principal component (PC1) was dominated by features associated with background wound media components (e.g., 660 and 732 cm^−1^), while the second principal component (PC2) showed strong contributions from the pyocyanin (415 and 545 cm^−^^1^), polysaccharide (865 and 1095 cm^−^^1^), and protein (1560 cm^−^^1^) peaks (Figure 5C,D). Together, PC1 and PC2 captured 22% and 20% of the total variance in the treated and non‐treated samples, respectively. A significant portion (≈80%) of the total variance remains distributed across higher‐order components, which likely reflects subtle biochemical variability within the biological matrix, along with residual measurement noise inherent to complex Raman datasets. This is typical for biological systems exhibiting heterogeneous molecular composition such as biofilms [71], where spectral variance is distributed across multiple low‐amplitude components rather than being concentrated in a small number of principal components. Crucially, the distinct separation of the temporal clusters within the PC1‐PC2 space demonstrates that the extracted signal remains statistically significant and distinguishable despite these underlying sources of variance. Consequently, key features from PC1 and PC2 loadings were subsequently used to quantitatively track the spatiotemporal dynamics of the biofilm secretions.

**FIGURE 5:**
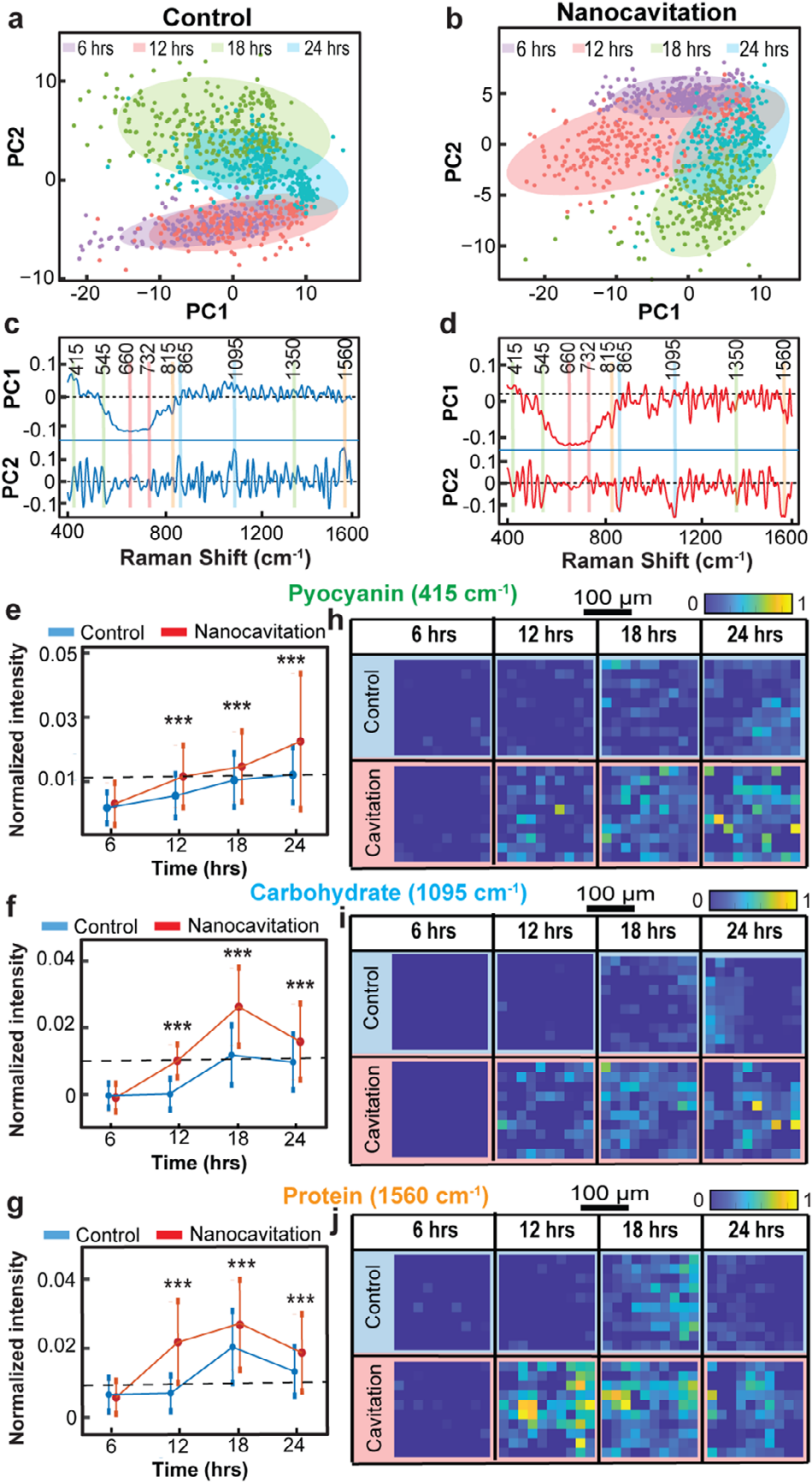
Multivariate spatiotemporal SERS analysis of P. aeruginosa biofilms. PCA scatter plots of temporally resolved SERS datasets from a) control and b) nanocavitation‐treated ABBA models interfaced with MPNMs show improved temporal separation in treated samples. c,d) Corresponding PC1 and PC2 loading plots identify the spectral features driving variance. e–g) Quantitative analysis of pyocyanin (415 cm^−^^1^), carbohydrate (1095 cm^−^^1^), and protein (1560 cm^−^^1^) peak intensities over time reveals significantly enhanced signal intensities in nanocavitation‐treated samples (***p < 0.001 (two‐sample t‐test); error bars represent standard deviation; *n* = 300 from three biological replicates; black dotted lines indicate noise level). h–j) Time‐resolved spatial maps of the same peaks visualize the spatiotemporal evolution of biofilm‐secreted components.

Quantitative tracking of the spectral markers revealed distinct temporal dynamics for the bacterial metabolites and matrix components. The pyocyanin peak at 415 cm^−^^1^ increased continuously with time, exhibiting statistically stronger signals in nanocavitation‐treated samples at 12, 18, and 24 h (*p* < 0.001) relative to controls, with mean values consistently above the noise threshold (Figure 5E). In contrast, the protein (1560 cm^−^^1^) and carbohydrate (1095 cm^−^^1^) peaks increased over the first 18 h and then saturated, again showing stronger signals in regenerated samples at all time points post‐inoculation (p < 0.001) (Figure 5F,G). Similar temporal trends were observed for the secondary 545 cm^−^^1^ pyocyanin peak and the 865 cm^−^^1^ carbohydrate peak (Figure S9). Collectively, these results confirm that nanocavitation regeneration improves molecular accessibility to SERS hotspots in wound media, enabling dynamic monitoring of biofilm growth with signals from secreted metabolites and EPS observed within 12 h.

Moreover, spatial intensity maps showed relatively uniform distributions of pyocyanin, protein, and carbohydrate signals in nanocavitation‐treated samples at later time points, whereas untreated controls exhibited sparse, clustered signals indicative of fouled hotspots (Figure 5E–G). To confirm the molecular origins of the observed peaks, we analyzed a *P. aeruginosa *transposon mutant of *phzM*, which is deficient in pyocyanin production (Figure S10). As expected, no peaks at 415 or 545 cm^−^^1^ were detected, validating their assignment to pyocyanin. In contrast, the carbohydrate (1095 cm^−^^1^) and protein (1560 cm^−^^1^) peaks were preserved and showed statistically significant enhancement in nanocavitation‐treated samples at later time points, consistent with improved hotspot accessibility (Figure S10).

## 3 Conclusion

Longitudinal, label‐free, and multi‐molecular monitoring within biofluids, particularly for in vivo applications, remains a major challenge but holds transformative potential for personalized disease management. Here, we demonstrate for the first time that plasmonic nanocavitation can locally regenerate nanogap SERS hotspots without structural or functional degradation over repeated cycles, enabling spatiotemporal molecular profiling at static biological interfaces for up to 24 h. This capability is enabled by MPNMs, which uniquely integrate wavelength‐multiplexed sensing (SERS) and actuation (nanocavitation) through engineered plasmonic nanogaps, while achieving seamless bio‐integration via a flexible, micro‐porous, and nanoprotruding architecture. The nanocavitation‐driven regeneration strategy significantly enhances SERS performance after 24 h exposure to human serum, achieving a LoD of 3.9 nm for pyocyanin compared with 2.0 µm for untreated controls, pushing sensitivity towards the lower end of clinically relevant concentrations (3–25 nm) reported in P. aeruginosa infected wounds.

As proof of concept for wound biofilm monitoring, we demonstrated in situ spatiotemporal profiling of P. aeruginosa biofilms using an in vitro wound model that mimics in vivo biofilm growth and the wound nutritional environment. At early biofilm stages (12 h), our MPNM platform detected P. aeruginosa‐specific metabolites (e.g., pyocyanin) and EPS components. Such timely detection is critical for diagnostic applications because early‐stage biofilms are more responsive to antimicrobial treatment than late‐stage biofilms [72, 73], allowing for more effective treatment. Importantly, the MPNM platform provides multi‐molecular spatiotemporal biofilm profiles (>10 molecular bands) within wound‐like environments encompassing secreted metabolites, EPS polysaccharides, and proteins. When coupled with machine learning analysis, these spectral insights can facilitate longitudinal extraction of clinically relevant spatial‐temporal information from wounds, such as biofilm species composition [74, 75], quorum‐sensing activity [76], physiological state (e.g., metabolic activity) [77], and EPS maturity‐level [78] in response to treatment. Collectively, these insights can support real‐time monitoring of treatment responses and personalized targeted therapy [79, 80], ultimately improving healing outcomes.

Looking forward, clinical translation requires overcoming several scientific and engineering hurdles outlined below. First, while the agarose‐based wound model effectively mimics nutrient diffusion and biofilm growth, we acknowledge that it does not fully capture the compositional and mechanical complexity of the dynamic wound bed. Therefore, the critical next step is to evaluate sensor performance and biosafety in in vivo models (e.g., murine wound models). Rheological discrepancies between the in vitro wound model and real wound exudate, specifically regarding viscosity and composition, could impact heat transport, nanobubble dynamics, and analyte diffusion. Additionally, the presence of host immune cells may compromise sensor performance through foreign body responses [81].

Second, the 24 h in vitro demonstration with 6 h measurement intervals illustrates the feasibility of nanocavitation‐mediated regeneration for certain short‐term chronic wound monitoring scenarios. For example, highly exudative wounds or wounds treated with certain dressing types (e.g., hydrogel or alginate dressings) commonly require dressing replacement every 1–3 d [82, 83], at which point a new sensor would be introduced. Consequently, continuous monitoring at clinically relevant intervals (e.g., every 6 h to track biofilm‐associated biochemical changes) could necessitate as low as four regeneration cycles over the operational lifetime of a single dressing. However, many chronic wound types, as well as other longitudinal monitoring applications beyond wound care, will require substantially longer deployment durations and/or higher sensing frequencies, necessitating a greater number of regeneration cycles. Consequently, future studies will be required to evaluate the effects of extended regeneration cycling on both device longevity and biosafety. Although controlled cavitation has been successfully employed in several in vivo biomedical applications, including ultrasound‐based lithotripsy [84] and blood‐brain barrier opening [85], and femtosecond lasers are already widely used in ophthalmology [86] and dermatological [87] procedures, the long‐term biological consequences of repeated plasmonic nanocavitation events remain to be established. In particular, the potential for cumulative tissue damage or inflammatory responses arising from repeated exposure to localized mechanical forces and transient thermal gradients warrants careful investigation. Addressing these questions will require systematic in vivo studies incorporating optimization of laser dose and regeneration frequency, real‐time temperature monitoring with closed‐loop laser dose control, histological evaluation, and longitudinal assessment of tissue responses. Such studies will be essential for establishing the long‐term safety and clinical viability of regenerative plasmonic sensing platforms.

Third, while this study focused on *P. aeruginosa*, clinical wounds are often polymicrobial. Extending this platform to complex mixed‐species biofilms or other disease contexts, such as cancer margins or implant infections, presents a computational challenge due to the spectral overlap of diverse biological components. Resolving specific signatures from 10+ co‐existing species in a real wound will require substantial advancements in the machine learning backends proposed here to achieve robust spectral deconvolution and effectively guide targeted therapeutic decisions [75]. Finally, hardware components such as the Raman spectrometer and compact ultrashort pulsed laser (e.g., sub‐nanosecond diode lasers) need to be integrated into a unified clinical platform. While compact, handheld Raman spectrometers are commercially available, miniaturizing the fs laser into a wearable patch remains a non‐trivial engineering barrier. Therefore, we envision the immediate application of this technology as a bedside diagnostic probe. In this realistic clinical workflow, a clinician would deploy the probe to the patient's bedside to perform sensor regeneration and measurement at defined intervals (e.g., every 6 h) to track infection dynamics. The resulting spatiotemporal maps of metabolite activity would then guide therapeutic decisions, such as the targeted debridement of high‐biofilm zones or the localized application of antimicrobials. If realized, this platform‐level technology, coupled with AI‐driven analytics, could enable real‐time diagnostics and closed‐loop personalized therapy in vivo, revolutionizing the management of chronic wound infections and extending to other life‐threatening diseases such as cancer.

## 4 Experimental Section

### 4.1 Fabrication of the MPNMs

The MPNMs were fabricated using a soft reverse nanoimprint lithography procedure, previously developed and documented [88, 89] and schematically depicted in Figure S1. Briefly, perfluoropolyether (PFPE) (Fluorolink PFPE, Solvay, Belgium) nanopillar array templates were first replicated from a silicon master mold (pillar diameter: 120 nm, height: 300 nm, periodicity: 400 nm) using UV nanoimprint lithography. The UV nanoimprint process involved curing under 2 bar pressure for 3 min, a second curing step under vacuum for 3 min, and post‐annealing at 100°C for 45 min. A 16% (w/w) solution of polymethyl methacrylate (PMMA) (molecular weight 15 000 g mol^−1^) was spin‐coated onto the PFPE nanopillar templates at 5000 rpm for 30 s. Solvent removal was achieved by baking at 180°C for 2 min, forming PMMA nanohole arrays on the PFPE template. These PMMA nanohole arrays were then transferred onto SU‐8 (SU‐8 2002, Kayaku Advanced Materials Inc., USA) microwell arrays (diameter: 16 m, height: 2 µm, periodicity: 64 µm) patterned on silicon wafers coated with an Omnicoat sacrificial layer (Kayaku Advanced Materials Inc., USA) via thermal reverse nanoimprint lithography (10 min, 2 bar, 170 °C). The SU‐8 microwell arrays were fabricated through masked photolithography (MA6 Mask Aligner, SUSS MicroTec, Germany). Residual PMMA was removed using reactive ion etching (RIE‐1C, Samco, Japan) with O_2_ plasma (30 sccm, RF power 30 W, 50 s), thereby opening the nanoholes. Subsequently, alternating layers of Au (25 nm) and SiO_2_ (8 nm and 12 nm from bottom) were deposited via electron beam evaporation (PVD250, Kurt J. Lesker, USA) through the PMMA nanohole mask. A 0.7 nm titanium adhesion layer was inserted between the Au and SiO_2_ layers, and a 1 nm chromium adhesion layer was deposited between the Au layer and the substrate. Following deposition, the PMMA mask was dissolved in anisole at 70°C. Nano‐protrusions were generated via reactive ion etching (O_2_ plasma, 30 sccm, RF power 30 W, 45 min). The dielectric nanogaps were subsequently opened by immersion in buffered oxide etchant (BOE 10:1, Transene Inc., USA) for 10 s at room temperature without agitation. To release the nanoplasmonic bio‐meshes, the devices were immersed in Remover PG (Kayaku Advanced Materials Inc., USA) for 15 min at 70°C. The released meshes were rinsed three times with deionized water using a Pasteur pipette to remove residual Remover PG, sterilized in ethanol for 30 min, and then transferred to target substrates using sterilized polyethylene terephthalate sheets as the transfer medium.

### 4.2 FDTD Simulations

A uniform 3 nm mesh was used in x‐, y‐, and z‐directions. The optical constants of Au were taken from Johnson and Christy. The Bloch boundary condition was used in x‐ and y‐directions with a periodicity of 400 nm, and the perfectly matched layer boundary condition was used in the z‐direction. The refractive indices of SiO_2_ and SU8 were set as 1.50 and 1.57, respectively.

### 4.3 Extinction Measurements

A UV–vis–NIR spectrophotometer (Cary 5000, Agilent, USA) was used to measure the extinction spectra.

### 4.4 Experimental Setup for Plasmonic Nanocavitation and Regeneration

All experiments were performed on a custom‐built microscopy setup. The nanocavitation pump source was a fs laser (Ultra II, Coherent, USA) tuned to 950 nm. Laser beams were made colinear using a dichroic mirror and focused onto the MPNM sample through the designated objective lens. The following fs laser irradiation parameters were used: 2 µs pulse repetition rate with fluence of 0.09 nJ µm^−2^. For nanobubble dynamics measurements (Figure 2B), back‐scattered light from a 780 nm CW probe laser was collected and directed to a high‐speed photodetector (PDA015A, Thorlabs, USA) connected to a high‐sampling‐rate oscilloscope (MOX4, Rohde&Schwarz, Germany) for time‐domain analysis and subsequent Fast Fourier Transform. For nanogap regeneration, the fs laser beam (beam size ≈ 13 µm^2^) firing at a 2 µs pulse repetition interval was raster‐scanned across the sample area. The sample was scanned in a bidirectional serpentine pattern at 1 mm s^−1^ along the x‐axis. After each line scan, the stage advanced by 5 µm in the y‐direction, and the scan continued in the reverse x‐direction. Under these conditions, each location on the sample received approximately 1000 pulses. Pump‐probe measurements were performed before each raster‐scan to ensure nanobubble generation. Safety interlocks and beam blocks were used to prevent unintended exposure.

### 4.5 SERS Measurements

For SERS measurements, a confocal Raman microscope (alpha 300RSA + , WItec, Germany) equipped with a 785 nm diode laser (Xtra II, Toptica, Germany) was used. The backscattered photons were detected with a spectrometer (UHTS300, WItec, Germany) equipped with a CCD camera (DU401A, Oxford Instruments, UK). Signals were collected using a 300 grooves mm^−1^ grating via a 20× objective lens with 1 mW power measured using an optical power meter (Thorlabs, Inc.).

### 4.6 SERS Data Analysis

Following signal acquisition, the acquired spectra were preprocessed using a built‐in software (Project v4.1 Software, WITec, Germany). The preprocessing steps included cosmic ray removal, background subtraction using the in‐built shape function, and Savitzky–Golay filtering to reduce high‐frequency noise while preserving spectral features (window size  =  5, polynomial order  =  3). SERS data were normalized using the plasmonic ERS signals, reflected by the pseudo peak at 86 cm^−1^, as the internal standard. Plasmon‐enhanced ERS signals from metal can serve as an internal standard for spatial and temporal calibration of molecular Raman scattering signals from analyte molecules at the same hotspots, enabling rigorous quantitative SERS analysis [54]. Spectra with ERS peak intensity below the elastic scattering peak intensity (0 cm^−1^) were eliminated. The noise level was determined as the standard deviation of the signal intensity within the silent spectral region at 2000 cm^−^^1^. The noise level was used as the baseline in SERS area maps. To statistically assess differences between measurements, independent two‐sample Student's t test was performed using MATLAB, with the resulting p‐value indicating the significance of the observed differences. PCA was performed in R using the prcomp function with both centering and scaling enabled. Mean‐centering ensures that principal components capture variations relative to the average spectrum, while scaling standardizes each variable to unit variance, preventing spectral regions with larger absolute intensities from disproportionately influencing the analysis. Raman wavenumbers from 390 to 1600 cm^−^^1^ were used as input variables.

### 4.7 Dual Analyte Regeneration Demonstration

A 5 µL droplet of 1 mm rhodamine 6G (R6G) (Sigma Aldrich, USA) in deionized water was drop‐cast onto the MPNM surface and allowed to dry at 60°C for 1 h. The substrate was then rinsed four times with deionized water and dried under a nitrogen stream to remove non‐adsorbed molecules. Subsequently, the substrate was immersed in a 10 µm pyocyanin (Sigma Aldrich, USA) solution in deionized water, after which three selected regions (100 µm × 100 µm each) were regenerated via raster scanning. SERS mapping was performed over a 500 µm × 150 µm area (100 × 30 pixels) encompassing both treated and untreated regions, with a scratch mark used as a marker to spatially align the SERS and nanocavitation measurements. An integration time of 0.1 s per pixel was used.

### 4.8 Pyocyanin Detection in Human Serum

To generate the zwitterionic surface layer, the substrates were incubated in a 100 µm aqueous solution of L‐cysteine (Sigma Aldrich, USA) for 1 h, then sequentially rinsed with ethanol and deionized water to remove unbound molecules. The functionalized substrates were then incubated in undiluted human serum (Sigma‐Aldrich, USA) for 24 h and gently rinsed with water. Pyocyanin solutions of varying concentrations (10^−^^9^–10^−^^5^ m in water) were subsequently added to the samples. A 4 mm^2^ area on each sample was regenerated via raster scanning, with untreated samples serving as controls. SERS mapping was then performed within the regenerated regions, with a 1 s integration time per pixel. Three technical replicated were performed.

A four‐parameter sigmoidal fit was applied to generate the calibration curve for the 410 cm^−1^ Raman peak intensity across varying pyocyanin concentrations using the following equation: $y=d+\frac{a-d}{1+{\left(\frac{x}{c}\right)}^{b}}$, where x = ‐log_10_(concentration of pyocyanin in M) and y = normalized Raman peak intensity. The fitted parameters were:

Nanocavitation‐treated: *a* = ‐0.028, *b* = ‐2.7325, *c* = ‐3.5690, and *d* = 0.0748
Untreated controls: *a* = ‐0.0041, *b* = ‐4.8594, *c* = ‐3.8087, and *d* = 0.0604

The LoD was determined using the standard approach of mean blank signal plus three times the standard deviation (mean_control_ + 3×SD_control_) as the threshold. Here, mean_control_ and SD_control_ correspond to the average intensity and standard deviation, respectively, of the 410 cm^−^^1^ peak intensity measured in pyocyanin‐free human serum samples (*n* = 25). The LoD was then obtained as the concentration at which this threshold intersects the fitted calibration curve.

### 4.9 In Vitro Wound Model

The in vitro wound model developed here is an amendment of the agar block biofilm assay [61], whereby wound‐like media [50% Bolton broth, 45% heparinized bovine serum (Hemostat Labs), 5% freeze‐dried laked horse red blood cells (Hemostat Labs)] [62] is solidified with 1% low melting point agarose. While molten the media was inoculated with either *P. aeruginosa* PAO1 or a phzM transposon mutant (phzM:: ISlacZ/hah) [90] at an OD 0.001 For microscopy experiments, biofilms were incubated for 24 h, stained with Syto9 (25 µm), and imaged on a Zeiss LSM880 confocal laser scanning microscope.

### 4.10 Regenerative Spatiotemporal SERS Measurements of the In Vitro Wound Model

Sterile MPNMs were placed on the agarose blocks. SERS measurements were conducted using a 20× objective lens with a laser power of 1 mW and an integration time of 1 s over a 200 µm × 200 µm area comprising 100 pixels. Measurements were taken at 0, 6, 12, 18, and 24 h. Nanocavitation treatment was performed prior to each SERS measurement between 6 and 24 h, while untreated samples served as controls. A 4 mm^2^ area on each sample was regenerated by raster scanning. Between measurements, the plates were incubated at 37°C. Principal component analysis (PCA) was performed in R, using Raman wavenumbers from 390 to 1600 cm^−^^1^ as input variables. Three biological replicates were analyzed.

## Author Contributions

A.G. and W.Z. conceived the study, with W.Z. supervising the project. A.G. fabricated the device and designed the experiments. A.G., Z.Z., and M.N. characterized device performance. M.N. set up the fs laser system and performed bubble generation experiments. E.M. conducted FDTD simulations. A.G., Z.Z., and M.N. carried out the biological experiments. E.S.G., S.E.D., and E.V.O. prepared and characterized the in vitro wound model. E.S.G. and P.V. provided feedback on the manuscript. A.G. and W.Z. wrote the manuscript with input from all authors. All authors approved the final version of the manuscript.

## Conflicts of Interest

The authors declare no conflicts of interest.

## Supporting information




**Supporting File**: advs76330‐sup‐0001‐SuppMat.docx.

## Data Availability

The data that support the findings of this study are available from the corresponding author upon reasonable request.
