# Supplementary material for: Femtosecond‐Laser Nanocavitation Regenerates SERS‐Active Plasmonic Nanogaps for Longitudinal Molecular Sensing at Biointerfaces
Source: Adv Sci (Weinh). 2026 Jul 3;13(53):e76330. doi: 10.1002/advs.76330 (PMC13334584; doi:10.1002/advs.76330)
Supplement: Supplementary file 1 [file ADVS-13-e76330-s001.docx]

Supplementary information

Femtosecond-laser Nanocavitation Regenerates SERS-active Plasmonic Nanogaps for Longitudinal Molecular Sensing at Biointerfaces

Aditya Garg, Ze Zong, Meitong Nie, Stacie E. Deaver, Elizabeth M. Van Order, Elieser Mejia, Peter Vikesland, Erin S. Gloag, and Wei Zhou*

A.G., Z.Z., M.N., E.M., and W.Z., Department of Electrical and Computer Engineering, Virginia Tech, Blacksburg, Virginia

24061, United States

A.G., Department of Mechanical Engineering, Massachusetts Institute of Technology, Cambridge, MA 02139, United States

S.E.D., E.M.V., and E.S.G., Department of Biomedical Sciences and Pathobiology, VA-MD College of Veterinary Medicine, Virginia Tech, Blacksburg, Virginia

24061, United States

P.V., Department of Civil and Environmental Engineering, Virginia Tech, Blacksburg, Virginia

24061, United States

*Email: wzh@vt.edu

**Figure S1:** Schematic illustration of the fabrication process to create multiresonant plasmonic nanoprotruding meshes (MPNMs).

**Figure S2:** A) Measured averaged surface enhanced Raman spectroscopy (SERS) spectrum of benenethiol molecules under 785 nm excitation. B) Histograms of the SERS enhancement factor (EF) measured using the 1070 cm^-1^ peak of benzenethiol, demonstrating a mean SERS enhancement factor of 10^7^. Details on the experiment and enhancement factor calculation can be found in our published works.^[1]^

**Figure S3.** Effect of fs laser fluence on nanogap regeneration performance. (A) SERS spectra acquired from nanocavitation-treated regions following irradiation with fs laser fluences below the bubble-generation threshold (0.03 nJ/µm²) and above the threshold (0.09, 0.105, and 0.12 nJ/µm²). Shaded regions represent the standard deviation. (B) SERS intensity maps of the R6G peak at 1504 cm⁻¹ and the pyocyanin peak at 415 cm⁻¹. White boxes indicate the nanocavitation treatment regions. From left to right, the treatment fluences are 0.03, 0.09, 0.105, and 0.12 nJ/µm². (C) Quantitative comparison of analyte signals demonstrating a statistically significant decrease in R6G peak intensity (1504 cm⁻¹) and increase in pyocyanin peak intensity (415 cm⁻¹) for fluences above the bubble-generation threshold relative to the 0.03 nJ/µm² condition (***p < 0.001, two-sample t-test). Error bars represent standard deviation (n = 60).

**Figure S4:** Schematic illustration of the working principle of the zwitterionic L-cysteine monolayer.

**Figure S5:** Violin plots show the 1350 cm⁻¹ pyocyanin peak and the 820 cm⁻¹ serum protein peak for the nanocavitation-treated samples and untreated controls across a range of pyocyanin concentrations. The error bars represent standard deviations, *** represents *p* values < 0.001 (two-sample t-test).

**Figure S6:** Pyocyanin detection limit of unfunctionalized substrates after 24 hour incubation in human serum. A) SERS spectra comparing unfunctionalized samples after a 24-hour incubation in human serum, followed by the introduction of pyocyanin at concentrations from 10⁻⁹ to 10⁻⁵ M. Shaded regions represent standard deviations. Green bars represent prominent pyocyanin peaks. B) Quantitative analysis of SERS signals. Violin plots show the 410 cm⁻¹ pyocyanin peak across a range of pyocyanin concentrations (error bars represent standard deviations; n=25; the red curve represents the four-parameter sigmoidal fit for calibration).

**Figure S7:** Average SERS spectra of Pseudomonas aeruginosa biofilms on lysogeny broth-agar plates measured using the MPNMs, showing clear pyocyanin signatures within 12 hours without the need for regeneration.

**Figure S8:** SERS spectrum of the wound media in the agar block biofilm assay (ABBA).

**Figure S9:** Quantitative analysis of pyocyanin (545 cm⁻¹) and protein (865 cm⁻¹) peak intensities over time reveals significantly enhanced signal intensities in nanocavitation-treated samples (***p < 0.001 (two-sample t test); error bars represent standard deviation; black dotted lines indicate noise level).

**Figure S10:** A) Time-resolved SERS spectra from 6 to 24 hours of biofilm growth of a *P. aeruginosa* mutant strain (ΔphzM) deficient in pyocyanin production. The spectra show the emergence of characteristic peaks from pyocyanin (green), proteins (orange), nucleic acids (red), and carbohydrates (blue) over time. Shaded regions represent standard deviations. B) Quantitative analysis of pyocyanin (415 and 545 cm⁻¹), carbohydrate (1095 cm⁻¹), and protein (1560 cm⁻¹) peak intensities over time (***p < 0.001 (two-sample t-test); error bars represent standard deviation; black dotted lines indicate noise level).

**Table S1**: Comparative Technology Analysis of SERS Antifouling and Regeneration Strategies

| **Technology / Strategy** | **Regeneration Mechanism** | **Invasiveness & Connectivity** | **Regeneration Time / Dynamics** | **Bio-compatibility & Stability** | **Key Limitations** |
| --- | --- | --- | --- | --- | --- |
| **Femtosecond Plasmonic Nanocavitation (MPNM)** *(This Work)* | **Active Thermomechanical:** Vapor nanobubble collapse generates intense shear stress (>100 kPa) and acoustic streaming to actively transport foulants. | **Non-invasive / Wireless:** Contact-free optical actuation through tissue-transparent windows; compatible with floating, flexible meshes. | **Microsecond Scale:** ~200 ns bubble lifetime allows rapid cleaning per pulse train; seconds–minutes for large-area raster scanning. | **High:** Biocompatible materials (SU-8, Au, SiO₂). Heat is confined to the nanoscale via ultrafast dissipation, preventing bulk tissue damage. | **Optical Access:** Requires optical transparency to the implant site; laser fluence must be managed to stay below tissue safety thresholds. |
| **Passive Antifouling^[2]^** *(Coatings, e.g., Zwitterionic)* | **Passive Repulsion:** Steric hindrance or formation of hydration layers (e.g., L-cysteine) to prevent protein adsorption. | **Non-invasive:** Surface chemistry modification only; no external trigger or power required. | **N/A (Finite Lifetime):** Delays fouling but provides no active regeneration mechanism. | **Moderate / Long-term Degradation:** Coatings are susceptible to oxidation and enzymatic degradation in complex biofluids. | **Irreversible Fouling:** Cannot regenerate once the coating fails; LoD degrades significantly (>2 µM) over time. |
| **Electrochemical Regeneration^[3]^** | **Active Desorption:** Application of oxidative/reductive potentials to strip adsorbed molecules. | **Minimally Invasive / Wired:** Requires wired electrodes integrated into the tissue interface, creating mechanical mismatch. | **Seconds – Minutes:** Dependent on potential cycling protocols. | **Low / Moderate:** Risk of generating reactive species harmful to tissue; rigid wiring may induce inflammation. | **Structural Damage:** Repeated cycling risks nanoscale restructuring of the sensor; lacks active mass transport leading to re-adsorption. |
| **Chemical / Photo-assisted^[4]^** *(Plasma, Photocatalysis, Electrokinetics)* | **Physical / Chemical Ablation:** High-energy plasma bombardment, ROS generation, or strong electric fields. | **Highly Invasive / External:** Plasma requires sensor removal (ex situ); Electrokinetics requires electrodes. | **Slow / Protocol-dependent:** Ex situ methods interrupt continuous monitoring. | **Incompatible:** Uses toxic solvents, generates harmful Reactive Oxygen Species (ROS), or requires destructive physical handling. | **Physiological Incompatibility:** ROS can harm host tissue ^4^; electrokinetics are often ineffective in high-ionic-strength biofluids due to Debye screening. |

**Table S2:** Comprehensive Optical and Laser Parameters

| **Parameter Category** | **Specification / Value** | **Unit** | **Rationale / Description** |
| --- | --- | --- | --- |
| **1. Nanocavitation Pump (Regeneration)** | | | |
| **Laser Source** | Femtosecond (fs) Pulsed | - | Ultrafast pulses localize energy deposition, minimizing bulk thermal loading. |
| **Center Wavelength** | 950 | nm | Tuned to the Magnetic Dipole (MD) resonance for efficient photothermal transduction. |
| **Repetition Rate** | 0.5 | MHz | 2 µs pulse spacing supports rapid cycling while allowing thermal relaxation between pulses. |
| **Fluence** | 0.09 | nJ/µm² | Set above cavitation threshold while avoiding plasmonic damage (e.g., melting/reflow). |
| **Pulse Energy** | ~1.17 | nJ | Calculated as Fluence × Beam Area (0.09 nJ/µm^2^ ×13 µm^2^). |
| **Beam Spot Size** | ~13 | µm² | Defines the spatial resolution of the regeneration area. |
| **Scanning Protocol** | 1 mm/s (x),  5 µm step (y) | - | Bidirectional serpentine raster for uniform coverage of the target region. |
| **Pulse Density** | ~1000 | pulses/spot | Provides cumulative cavitation events per pixel to remove persistent foulants. |
| **2. Pump–Probe (Validation)** | | | |
| **Probe Wavelength** | 780 | nm | Continuous-wave probe used to monitor transient back-scattering associated with nanobubble formation and collapse; selected near the electric-dipole (ED) resonance to maximize scattering sensitivity during cavitation events. |
| **Readout Mode** | Back-scattering | - | Captured by high-speed photodetector/oscilloscope for time-domain reconstruction. |
| **Observed Lifetime** | ~200 | ns | Confirms transient nanobubble formation and rapid energy dissipation. |
| **3. SERS Sensing (Readout)** | | | |
| **Excitation Wavelength** | 785 | nm | Tuned to the ED resonance for maximal SERS enhancement. |
| **Laser Mode** | Continuous Wave (CW) | - | Stable excitation for Raman acquisition. |
| **Power at Sample** | 1 | mW | Low-power readout to mitigate photothermal/photochemical perturbation of analytes (measured at the sample via 20× objective). |
| **Integration Time** | 0.1 – 1.0 | s | Adjusted for signal strength; supports repeated, longitudinal measurements. |

**Note: The actuation laser employs femtosecond pulse durations. While the precise pulse width is source-dependent (e.g., Coherent Ultra II), the “fs” designation typically corresponds to ~100–300 fs, enabling ultrafast energy deposition that favors highly localized heating and minimizes macroscopic thermal diffusion.*

**Table S3:** 1504 cm^-1^ R6G SERS peak intensity statistics over 5 regeneration cycles.

| **Cycle** | **Condition** | **n** | **Mean** | **SD** | **RSD** |
| --- | --- | --- | --- | --- | --- |
| 1 | R6G | 898 | 11.3111 | 3.6555 | 32.32% |
| 1 | Clean | 893 | 6.5497 | 3.1777 | 48.52% |
| 2 | R6G | 899 | 11.1811 | 3.8799 | 34.70% |
| 2 | Clean | 900 | 7.6469 | 3.6287 | 47.45% |
| 3 | R6G | 899 | 9.7710 | 3.2847 | 33.62% |
| 3 | Clean | 900 | 7.7179 | 3.2831 | 42.54% |
| 4 | R6G | 891 | 10.5737 | 3.7308 | 35.29% |
| 4 | Clean | 900 | 7.7114 | 3.4398 | 44.61% |
| 5 | R6G | 899 | 10.5152 | 3.7318 | 35.49% |

**Table S4:** Raman peak assignments.

| **Raman shift (cm^-1^)** | **Origin** | **Peak assignment** |
| --- | --- | --- |
| 410-415 | Pyocyanin | In plane ring deformation^[5]^ |
| 545 | Pyocyanin | In plane ring deformation^[5]^ |
| 660 | Guanine | Ring breathing modes^[6]^ |
| 732 | Adenine | In plane ring breathing^[6]^ |
| 815-822 | Proteins | Tyrosine (Ring breathing)^[6]^ |
| 865 | Polysaccharides (e.g., alginate) | C–C str, C–O–C 1,4-glycosidic link^[6, 7]^ |
| 995-1013 | Proteins | Phenylalanine^[6]^ |
| 1035-1045 | Proteins | Phenylalanine^[6]^ |
| 1065 | Proteins/lipids | C-N stretch |
| 1095 | Polysaccharides (e.g., alginate) | C–C str, C–O–C 1,4-glycosidic link^[6, 7]^ |
| 1180 | Proteins/Nucleic acids | CH bending (tyrosine)/cytosine and guanine ^[6]^ |
| 1250 | Proteins | Amide III^[6]^ |
| 1350 | Pyocyanin | Ring stretching^[5]^ |
| 1560 | Proteins | Tryptophan/Tyrosine/Amide II^[6]^ |

**Supplementary Text 1:** **Biocompatibility of the Multiresonant Plasmonic Nanoprotruding Mesh (MPNM) Platform**

The biocompatibility of the Multiresonant Plasmonic Nanoprotruding Mesh (MPNM) platform and its suitability for long-term bio-interfaced molecular analysis arise from the synergistic interplay of (i) tissue-like mechanical compliance, (ii) a macroporous structural architecture that preserves mass transport, (iii) the long-established *in vivo* tolerance of the constituent materials, and (iv) a rigorous operational safety profile governed by fundamental physical limits. Mesh-structured biointerfaces with analogous geometries have supported stable chronic neural recordings *in vivo* for months in rodent brain models, demonstrating that ultrathin, porous, mechanically compliant implants can reduce tissue disruption and sustain functional biointegration over extended timescales.^[8, 9]^

- **Tissue-Like Mechanical Properties:** The MPNMs incorporate a highly flexible, ultrathin, and open mesh geometry that yields low bending stiffness and reduced interfacial mechanical mismatch with soft tissue. A reduced mechanical mismatch is important because large differences in stiffness between an implanted device and surrounding tissue can increase micromotion-induced strain, which can amplify local inflammation and fibrotic encapsulation.^[9, 10]^
- **Macroporous Structural Architecture:** The macroporous mesh architecture provides continuous fluidic pathways through and around the mesh openings, which helps preserve diffusive and convective transport of oxygen, nutrients, and soluble signaling molecules across the biointerface. Maintaining mass transport is critical for maintaining homeostasis at the wound interface, as restricted transport near an implanted surface can locally deplete resources and promote adverse foreign-body responses.^[9]^
- **Material Biocompatibility and Surface Chemistry:** The MPNM is composed of well-established biocompatible materials, including SU-8 (an epoxy-based negative photoresist), gold (Au), and silicon dioxide (SiO₂), all of which have demonstrated long-term *in vivo* tolerance.^[8, 9]^ Gold contributes to biocompatibility because it is chemically inert under physiological conditions, while SiO₂ provides a stable, insulating barrier. Additionally, the functionalization layer, L-cysteine (L-Cys), is a naturally occurring amino acid known for its low immunogenicity, which helps preserve the biological inertness of the device.

**Supplementary Text 2:** **Dual analyte experiments at varying laser fluences.**

The same experimental setup described in Figures 2D–F was employed. Four predefined regions were raster scanned using a fs laser at fluences below the bubble-generation threshold (0.03 nJ/µm²) and above the threshold (0.09, 0.105, and 0.12 nJ/µm²), followed by SERS mapping. Bubble generation was confirmed through pump-probe measurements. Figure S3A presents the SERS spectra acquired from the fs laser treated regions following irradiation at the different laser fluences. For fluences above the bubble threshold, the intensities of the R6G peaks (607, 782, 1185, 1309, and 1504 cm⁻¹) decreased after treatment, indicating the removal of R6G molecules from the nanogap hotspots (Figure S3B). In contrast, an increase in the R6G signal was observed in the region treated below the bubble threshold. This behavior may arise from a combination of limited molecular desorption, photobleaching-induced reduction of R6G fluorescence background that improves the Raman signal-to-noise ratio, and the re-deposition of desorbed R6G molecules into the nanogaps through thermally driven convective flow. In the absence of nanocavitation, these molecules are not subjected to the strong mechanical forces required to transport them away from the substrate. Quantitatively, the R6G peak intensity was approximately 1.9-fold lower for fluences above the bubble threshold than for the sub-threshold condition (Figure S3C).

Simultaneously, the pyocyanin peaks (415, 547, and 1615 cm⁻¹) increased in intensity under all treatment conditions, consistent with improved molecular access to the regenerated hotspots (Figure S3B). However, the enhancement was substantially greater above the bubble threshold, where the pyocyanin peak intensity was approximately 2.1-fold higher than that observed below the threshold (Figure S3C), demonstrating a marked improvement in regeneration performance upon the onset of nanocavitation. Notably, increasing the laser fluence from 0.09 to 0.12 nJ/µm² did not result in any significant additional decrease in R6G intensity or increase in pyocyanin intensity, despite the greater thermal energy deposited at these higher fluences (Figure S3C). Collectively, these results indicate that nanogap regeneration is strongly driven by mechanical forces generated during nanocavitation rather than by thermal effects alone.

**Supplementary Text 3: Effects of pH on L-cysteine antifouling.**

pH is another important consideration for zwitterionic antifouling compounds such as L-cysteine. Our experiments were conducted in human serum (pH 7.3–7.5) and in an in vitro Pseudomonas aeruginosa biofilm model, where local acidification due to extracellular matrix components, including extracellular DNA, may reduce the pH to approximately 4–6.^[11]^ Across this pH range (4–7.5), L-cysteine remains zwitterionic, in which the thiol group binds to gold, the carboxyl group remains deprotonated (–COO⁻), and the amine group remains protonated (–NH₃⁺), enabling antifouling performance.

**Reference:**

[1]	J. Song, W. Nam, W. Zhou, Advanced Materials Technologies 2019, 4, 1800689.

[2]	F. Sun, H.-C. Hung, A. Sinclair, P. Zhang, T. Bai, D. D. Galvan, P. Jain, B. Li, S. Jiang, Q. Yu, Nature communications 2016, 7, 13437; Z. Song, Y. Li, H. Teng, C. Ding, G. Xu, X. Luo, Sensors and Actuators B: Chemical 2020, 305, 127329; H. Durand, A. Whiteley, P. Mailley, G. Nonglaton, ACS Applied Bio Materials 2022, 5, 4718.

[3]	S. M. Sibug-Torres, D.-B. Grys, G. Kang, M. Niihori, E. Wyatt, N. Spiesshofer, A. Ruane, B. de Nijs, J. J. Baumberg, Nature Communications 2024, 15, 2022.

[4]	X. Jiang, X. Sun, D. Yin, X. Li, M. Yang, X. Han, L. Yang, B. Zhao, Physical Chemistry Chemical Physics 2017, 19, 11212; M. Chen, W. Luo, Z. Zhang, R. Wang, Y. Zhu, H. Yang, X. Chen, ACS applied materials & interfaces 2017, 9, 42156; M. Viehrig, S. T. Rajendran, K. Sanger, M. S. Schmidt, T. S. Alstrøm, T. Rindzevicius, K. Zór, A. Boisen, Analytical chemistry 2020, 92, 4317.

[5]	G. Bodelón, V. Montes-García, V. López-Puente, E. H. Hill, C. Hamon, M. N. Sanz-Ortiz, S. Rodal-Cedeira, C. Costas, S. Celiksoy, I. Pérez-Juste, Nature materials 2016, 15, 1203.

[6]	Z. Movasaghi, S. Rehman, I. U. Rehman, Applied Spectroscopy Reviews 2007, 42, 493.

[7]	N. P. Ivleva, M. Wagner, H. Horn, R. Niessner, C. Haisch, Analytical and bioanalytical chemistry 2009, 393, 197.

[8]	G. Hong, T.-M. Fu, M. Qiao, R. D. Viveros, X. Yang, T. Zhou, J. M. Lee, H.-G. Park, J. R. Sanes, C. M. Lieber, Science 2018, 360, 1447; T.-M. Fu, G. Hong, T. Zhou, T. G. Schuhmann, R. D. Viveros, C. M. Lieber, Nature methods 2016, 13, 875.

[9]	T. Zhou, G. Hong, T.-M. Fu, X. Yang, T. G. Schuhmann, R. D. Viveros, C. M. Lieber, Proceedings of the National Academy of Sciences 2017, 114, 5894.

[10]	Z. Z. Aditya Garg, Peter Vikesland, Erin S. Gloag, and Wei Zhou, npj biosensing 2025.

[11]	N. Mozaheb, P. Rasouli, M. Kaur, P. Van Der Smissen, G. Larrouy-Maumus, M.-P. Mingeot-Leclercq, Microbiology spectrum 2023, 11, e04832.
